# Allergic diseases of the skin and drug allergies – 2001. Amdosol – a non-steriod drug for the treatment of chronic atopic-dermatitis

**DOI:** 10.1186/1939-4551-6-S1-P91

**Published:** 2013-04-23

**Authors:** Vishwanath Kundur, Mam Jamakhandi

**Affiliations:** 1 Allergy and Asthma Center, Dharwar, India; 2 Taaha Chemicals, Hubli, India

## Background

To show that Amdosol, a non-steroid synthetic compound, in an ointment form, can effectively control the signs and symptoms of Chronic Atopic Dermatitis (CAD) in 2-3 weeks without causing any side effects. Amdosol ointment (AO) contains Amdosol a white crystalline chemical compound, in white petrolatum. Clinical and animal studies have shown that AO has no side effects.

## Methods

### Animal study

12 healthy albino rabbits were subjected to Draize Skin Test. Twice daily applications of AO on intact abraded skin of rabbits, up to 2gm/Kg wt for a week. None of the rabbits showed any erythema or eschar formation and no irritation. Ref: Study done at M.S.Ramaiah Drugs and Allied Products Testing Center, Bangalore.

### Clinical study

Ten patients of CAD selected for study.

### Selection criteria

1. Personal and family history.

2. Appearance of the skin: Dry itchy red skin, swelling, blistering, oozing, crusting & scaling.

3. Typical distribution of lesions.

4. Chronically relapsing.

5. Associated Allergies – Asthma, All-Rhinitis

Prior to the present study, all patients were treated with Steroid ointments with no relief.

### Dosage and administration

Twice daily application of AO on affected parts of the patients for 2-3 weeks. Total of 30-60gms of AO was used on each patient depending on severity and extent of lesions. No other drugs - oral or topical, or particular diet was advised.

## Results

• Signs and symptoms fully controlled and lesions healed in 2-3 weeks.

• No recurrence or side effects observed in all the patients.

• Systemic absorption of Amdosol was not detected in man.

## Conclusions

Amdosol a non-steroid ointment effectively controlled signs & symptoms of Chronic Atopic Dermatitis (CAD) and healed the lesions in 2-3 weeks. Importantly AO has no side effects unlike steroid ointments presently in use to treat CAD. AO is effective in the treatment of CAD of any aetiology. Amdosol is not only anti-inflammatory, but also has antibacterial and antifungal property. Studies performed with AO indicate that it is in the super-high range of potency compared with other topical steroid ointments. Amdosol ointment can possibly revolutionize the treatment of Chronic Atopic Dermatitis

